# Fascia lata packing and tension suturing for symptomatic pseudomeningocele after recurrent cervical intradural tumour resection

**DOI:** 10.1038/s41598-021-84193-x

**Published:** 2021-03-02

**Authors:** Huanbo Xu, Yangliang Huang, Yi Zhong, Guowang Lu

**Affiliations:** 1 Department of Neurosurgery, The First Affiliated Hospital of Shantou University Medical College, Shantou, 515000 China; 2 Department of Spine Surgery, The First Affiliated Hospital, Sun Yat-Sen University, Guangzhou, 510700 China; 3 Department of Physiology, Guangzhou Medical University, 195 Dongfeng Xi Road, Guangzhou, 510000 China

**Keywords:** Diseases, Medical research

## Abstract

In recurrent posterior cervical intradural tumour resections, serious complications can be developed. The dural can become affected by inflammatory factors or removed during tumor resection; if cerebrospinal fluid (CSF) leakage cannot be stopped by duraplasty, artificial meninges or fascia repair, large pseudomeningocele can develop posteriorly within the soft tissue of the neck. When the pressure of the CSF cannot be maintained steadily, persistent clinical symptoms can occur, such as postural headache or central fever. Moreover, the skin can also be penetrated in a few patients even after extension of the drainage duration, lumbar cistern drainage or skin suturing, leading to the induction of life-threatening intra-cranial infections. Is there a simple and effective surgical method to address this scenario? The aim of this study was, therefore, to investigate the effectiveness of fascia lata packing and tension suturing in the treatment of symptomatic pseudomeningocele after recurrent posterior cervical intradural tumour resection. In our study, nine consecutive spinal surgery patients were recruited from January 2008 to January 2018. All pseudomeningoceles were combined with postural headache, central neurological fever or wound non-union. There were 3 cases of melanocytoma, 3 cases of nasopharyngeal carcinoma metastasis, 2 cases of breast cancer metastasis, and 1 case of spinal canal lymphadenoma. Standard patient demographics, diagnosis, post-operative symptoms, wound healing time, and the largest pre- and last follow-up pseudomeningocele area on axial MRI sections were recorded. All cases were followed-up successfully, from 12 to 24 months, with an average of 15.3 months. Our observations indicate that all wounds healed successfully. The wound union time was 20.7 days on average. After wound union, these patients became symptom free. The largest cerebrospinal fluid area on axial MRI sections improved significantly from 42.9 ± 5.01 cm^2^ at p re-operation to 6.6 ± 1.89 cm^2^ at 1 year post-operation (*P* < 0.05); Our data indicate that .the proposed procedure is simple, safe and effective. And more importantly, it allows rapid closure of any cerebrospinal fluid leakage pools.

## Introduction

Pseudomeningocele refers to the collection of cerebrospinal fluid (CSF) in a fibrous capsules which are located in spaces between subcutaneous tissues^1, 2^. However, few studies have been conducted on these complex formations^3^. Fortunately, with the development of dural repair techniques, such as duraplasty, artificial meninges or fascia replacement, complications such as pseduomeningocele are becoming increasingly unusual. However, such complications can occasionally be encountered in recurrent posterior cervical intradural tumour resection. Three major factors have been considered for the emergence and enlargement of pseudomeningoceles. First, the invasive growth patterns of sub-dural tumours and inflammatory infiltration of the dura often result in a difficult dural repair^4^. Second, after total laminectomy and internal fixation, cervical muscles that detach from the cervical lamina will undergo atrophy^5^; Third, repeated incisions would decrease the ability of the skin to heal. If the dural defect is allowed to persist for a few weeks, a large pseudomeningocele can form^6^. Even after extension of the drainage duration, lumbar cistern drainage or skin suture are employed, persistent clinical symptoms, such as postural headache or central fever, could persist for some patients^7–9^. Furthermore, the skin could be penetrated under increased pressure from the CSF, and would produce life-threatening intra-cranial infections^10^.

Since mainstream techniques have been shown to be ineffective, how can these patients be treated? Indeed, these techniques focus on dural repair while ignoring one way to solve the above mentioned problems: increasing the soft tissue volume. This hypothesis has not been previously studied. Therefore, we invented a method involving fascia lata packing and tension suturing. In this method, no water-tight sutures are employed; the fascia lata is packed inside the large pseudomeningocele with tension sutures to increase the soft tissue volume and ensure that the fascia contacts and fuses with the surrounding tissue. This is considered the last resort for symptomatic pseudomeningocele when every rescue method is ineffective. To our knowledge, this method has not been reported before.

## Results

### Patient characteristics

From January 2008 to January 2018, the fascia lata packing and tension suturing procedure was applied to a total of 9 patients. Among them, there were 6 males and 3 females. With the average age of 47.4 years. Six of these patients had previously received chemotherapy, and radiotherapy patients were excluded. All patients presented with normal liver and kidney functions. There were 3 cases of melanocytoma, 3 cases of nasopharyngeal carcinoma metastasis, 2 cases of breast cancer metastasis, and 1 case of spinal canal lymphadenoma. All of them complained of postural headache and central neurological fever, while 3 of them were complicated by cutaneous fistulae. Four patients consulted for tumour recurrence, and 5 patients consulted for pre-existing pseudomeningocele. For tumour recurrence, revision surgery included additional laminectomy, internal fixation and tumour resection. Furthermore, all these patients received dural repair treatments, such as duraplasty, artificial meninges or fascia replacement. Unfortunately, the rescue mission was not successful, and pseudomeningocele developed. Conservative treatment were then administered as the first choice for all patients, including extension of the drainage duration, lumbar cistern drainage or skin suturing. Surgery was offered when the conservative treatments failed and symptoms lasted for 3 weeks or when cutaneous fistulae were observed.

**Figure 1:**
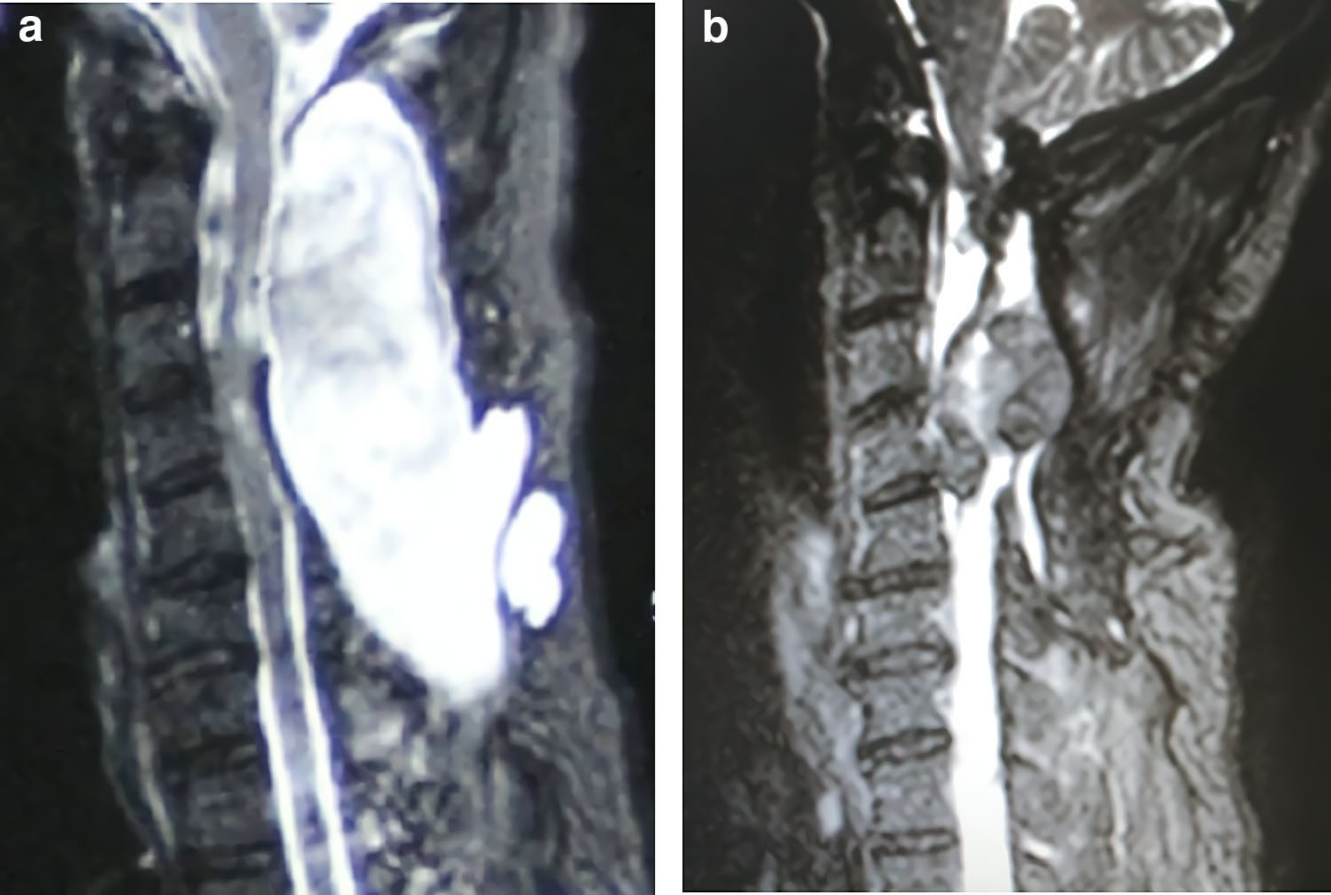
The first case. (**a**) Pre-operative area of the pseudomeningocele on sagittal MRI section. (**b**) Post-operative area of the pseudomeningocele on sagittal MRI section.

**Figure 2:**
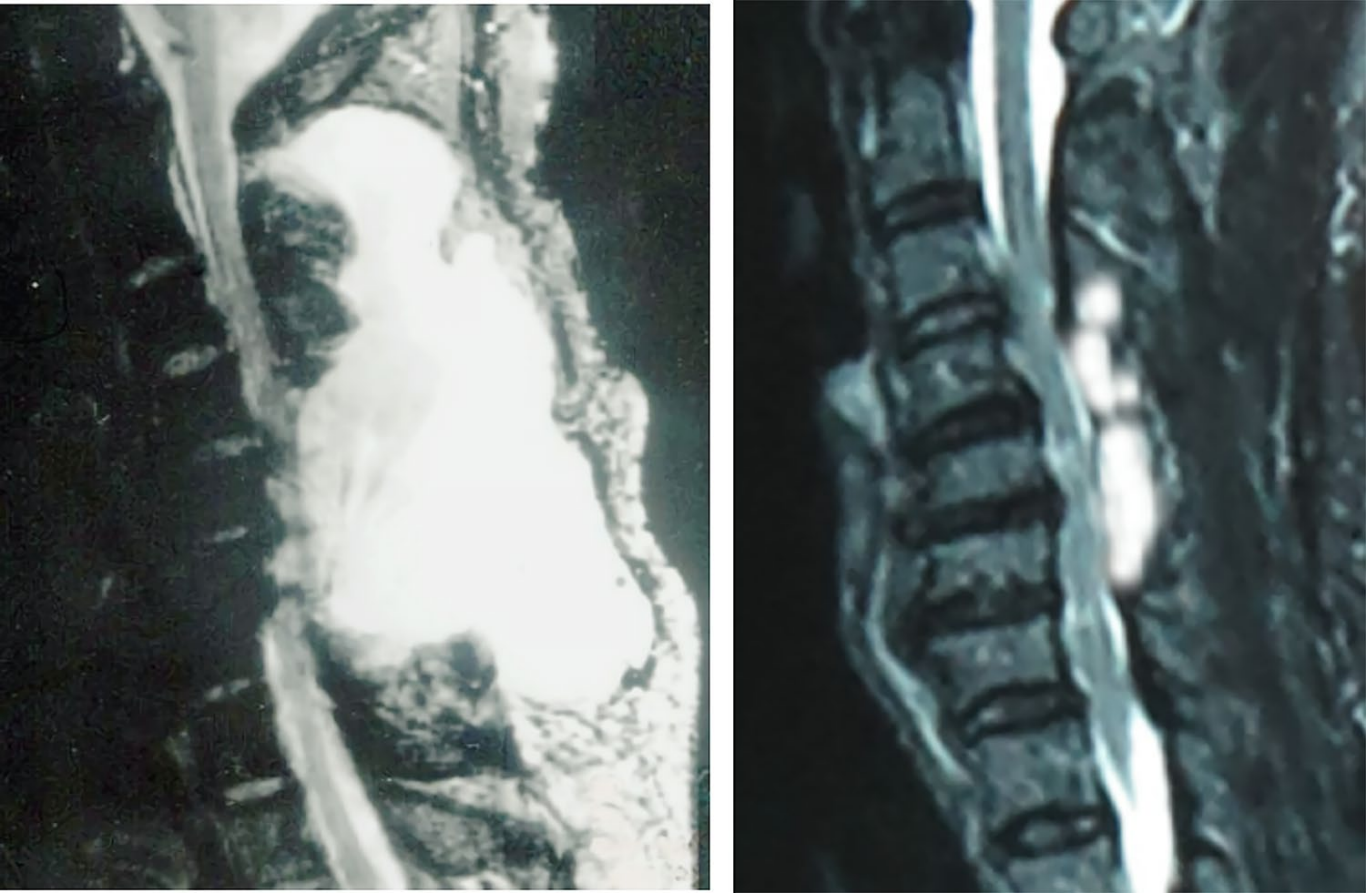
The second case. (**a**) Pre-operative area of the pseudomeningocele on sagittal MRI section. (**b**) Post-operative area of the pseudomeningocele on sagittal MRI section.

Patients were successfully followed-up for 15.3 months on average (12–24 months). On average, all wounds were successfully healed by 20.7 days post-operation on average. After all sutures were removed, the postural headache and central neurological fever were alleviated. The area of the pseudomeningocele on sagittal MRI section improved from 42.9 ± 5.01 cm^2^ at pre-operation to 6.6 ± 1.89 cm^2^ 1 year post-operation and the this difference was statistically significant (*P* < 0.05) (Table 1) (Figs. 1, 2).

**Table 1:** Patient demographics.

No	Age	Sex	Diagnosis	Initial surgery	Chemotherapy	Second/last surgery	Time between last two surgeries (days)	Combined symptoms	CSF leakage area (cm^2^)		Follow-up time
									Pre-op	Follow-up	
1	48	M	Melanocytoma	C3-5 laminectomy and tumour resection	None	C2 total laminectomy with internal fixation	18 s	Postural headache central neurological fever	40.9	5.2	24
2	29	F	Breast cancer	C3-6 laminectomy and tumour resection	TP	C2 total laminectomy with internal fixation	23 s	Postural headache central neurological fever and cutaneous fistulas	37.3	4.8	12
3	51	M	Nasopharyngeal carcinoma	C4-6 laminectomy and tumour resection	TPF	C3 partial laminectomy	18 s	Postural headache central neurological fever	38.5	7.5	18
4	45	M	Nasopharyngeal carcinoma	C2-6 laminectomy, internal fixation and tumour resection	TPF	C7 laminectomy and internal re-fixation	22 s	Postural headache, central neurological fever and cutaneous fistulas	48.2	5.9	18
5	71	M	Lymphadenoma	C2-6 laminectomy, internal fixation and tumour resection	8R-6/8CHOP21	C7 laminectomy and internal re-fixation	21 s	Postural headache central neurological fever	39.2	7.3	18
6	36	M	Melanocytoma	C3-5 laminectomy and tumour resection/C2 partial laminectomy with internal fixation	None	C2 total laminectomy with internal re-fixation	20 s	Postural headache, central neurological fever and cutaneous fistulas	51.1	6.1	12
7	59	F	Breast cancer	C4-5 laminectomy and tumour resection	TP	C6 laminectomy and internal fixation	18 s	Postural headache central neurological fever	48.5	4.5	12
8	28	F	Melanocytoma	C4-7 laminectomy and tumour resection and internal fixation	None	T1 laminectomy with internal re-fixation	17 s	Postural headache central neurological fever	40.8	10.7	12
9	60	M	Nasopharyngeal carcinoma	C3-5 laminectomyand tumour resection	TPF	C2laminectomy with internal fixation	18 s	Postural headache central neurological fever	41.6	7.1	12
Average ± (SD)	47.4	N.A	N.A	N.A	N.A	N.A		N.A	42.9 ± 5.01*	6.6 ± 1.89*	15.3

*There was a significant difference between these two measurements, *P* < 0.05.

### Complications

Pain at the donor site after exercise was reported by 4 patients, and cervical pain induced by the tension sutures was reported by 3 patients. The pain was temporary and could be relieved by NSAIDs. Although tumour recurrence was observed in 4 patients. We did not observe pseudomeningocele recurrence.

### Typical case

A 32-year-old man complained of numbness in both upper limbs for 2 months and underwent C3-5 laminectomy for resection of a cervical neoplasm at the local hospital. His histopathological diagnosis was melanotic schwannoma. Three years later, the numbness recurred with muscle weakness in both upper limbs. Pre-operative MRI revealed an extramedullary intra-dural tumour with compressed the spinal cord dorsally at the axis level. Additional C2 partial laminectomy with internal fixation from C2-5 was performed. The second histopathological diagnosis was melanocytoma. After the surgery, he regained his muscle strength and daily living activities. However, 1 year later, he was referred to our department for the second time for symptom recurrence and for internal fixation failure. Upon hospital admission, his chief complaints were numbness and weakness of both the upper and lower limbs. Therefore, surgical posterior decompression of the total axis lamina and refixation were performed. The dura was thin and expanded, and after tumour resection, it was repaired with artificial meninges. The wound was closed in the usual multi-layered fashion. Post-operation, there were repeated bouts of postural headache and central neurological fever after drainage was removed. Twenty days post-operation, the wound was penetrated by CSF pressure. Fascia lata packing and pressure suturing were performed. After 1 year of follow-up, the CSF leakage pool had closed almost completely (Fig. 1).

## Discussion

As duraplasty, artificial meninges and fascia repair have been effective for treating cerebrospinal fluid leakage, symptomatic iatrogenic pseudomeningocele have become rarer^11, 12^. However, the development of the medical system in China is not progressing equally throughout the country. Cervical tumour resection and CSF leakage rescue techniques have not been mastered well by every surgeon; as a result, tumour recurrence and pseudomeningocele were still encountered more frequently than expected. As one of the largest hospitals in southern China, we receive patients referred from 6 provinces. From January 2008 to January 2018, 9 patients consulted our services for revision surgery for recurrent tumour resection or symptomatic pseudomeningocele treatment. For tumour resection, we attempted to resect the tumour completely and repair the dura with duraplasty, artificial meninges or fascia repair. However, inflammatory reactions and destruction from the tumour left the dura thin and fragile. Repairing the dura with a water-tight suture was difficult.

Post-operatively, when the drainage indicated obvious CSF leakage, extension of the drainage duration and intermittent clamping of the drainage tubes were performed. If the leakage could not be stopped, skin suturing was employed after removal of the drainage. Typically, there would be no clinical symptoms following these procedures^13^. However, some patients would develop symptoms, such as postural headache or central neurological fever. The treatment principle was to reduce the pressure and to promote healing of the dura, such as with lumbar cistern drainage^14^. In our experience, some of these patients developed persistent symptomatic pseudomeningocele, and the odds of skin penetration were high when the symptoms persisted for 3 weeks. Thus, re-operation would be considered to relieve the symptoms at this point.

For patients who consulted our services for pseudomeningocele treatment, conservative treatment was the first choice. However, if the symptoms lasted for 3 weeks or there were pre-existing cutaneous fistulae, surgery was offered. Similar to extracorporeal membrane oxygenation for pneumonia, fascia lata packing and tension suturing were considered the final step for symptomatic pseudomeningocele when every other rescue method became ineffective. Although the number of cases in our study is small, all of the patients who received the procedure had satisfactory clinical outcomes.

The current dural repair methods have been focused onto stopping the CSF leakage with water-tight sutures and fibrin glue. These extraordinary steps are taken to ensure a watertight seal using suturing as the basic technique and restricting water in the sub-dural space^9, 15^. Additionally, use of collagen matrix is currently popular in dural repair; it is particularly indicated when primary suture closure may not be feasible. However, sutures are still required for the repair of intra-dural tumours when the defect is large^16–18^. It has become clear that even these obsessive attempts do not always prevent CSF leaks, resulting in a 5–10% failure rate^19–22^. Without question, watertight techniques should have a higher failure rate for recurrent cervical tumour resections.

Is there another way to solve these problems when dural repair techniques and conservative treatments are ineffective? Based on our knowledge, soft tissue atrophy of the posterior cervical muscle is another important factor in the formation of pseudomeningocele. Therefore, increasing the amount of soft tissue should be a good way to solve these problems. However, when detached from the cervical laminae after total laminectomy or internal fixation, the muscles would undergo atrophy, and if the pressure from the CSF increases further, a large pseudomeningocele can be produced within the empty space of the neck. Since the muscles of the thoracic and lumbar spine are stronger than the cervical muscles, large pseudomeningoceles are less likely to develop among these soft tissues^23^. In other words, these muscles have a self-packing effect, and it is difficult for a large capsuleto form when there are abundant soft tissues occupying the space. Thus, when duraplasty, artificial meninges, fascia repair and collagen matrix are ineffective^24^, increasing the amount of soft tissue should be usefull for treatment of pseudomeningocele.

Among our treated patients, tumour recurrence occurred in 4 but none of them developed pseudomeningocele recurrence. Thus, we hypothesize that soft tissue atrophy may be more important than dural defects in the formation and enlargement of pseudomeningoceles^25, 26^. To our knowledge, this is the first report about treating pseudomeningocele by increasing the amounts of soft tissues, while other methods focus on repairing the dura. Furthermore, the implanted fascia was not sutured to the dura but rather packed into the space of the pseudomeningocele and fused with the posterior soft tissue with two sets of tension sutures. The total operational time was no more than half an hour, and no obvious bleeding occurred. Thus, the surgical procedure was less demanding than that of other methods, and it was simple, safe, effective, and suitable for patients with malignant or recurrent tumours.

Fascia lata packing and tension suturing offers an alternative choice for treating pseudomeningocele. In this procedure, three approaches were utilized to eliminate the cavities of the pseudomeningoceles. First, by packing the cavity with fascia lata, the dead space produced by the pressure of CSF was eliminated. Second, since the fascia lata and posterior cervical soft tissue are muscle-ligament compounds, the compatibility between them is excellent; in other words, they can be easily integrated with each other^27, 28^. Third, when the abdominal closure needles are passed through the posterior cervical tissue and fascia lata, the lateral walls of pseudomembranes and implanted fascia lata can be secured with each other, thus ensuring good blood supply and preventing fascia displacement.

Among our patients, no neurological defects induced by spinal cord compression were observed. First, the implanted fascia were pieces of soft tissue which were not strong enough to produce compression events, except if there was soft tissue accumulation. However, such accumulation was not impossible when the fascia is fixed with the surrounding tissue with two sets of tension sutures. Second, after the mid-line sutures were removed at 14 days post-operation, the tension sutures were also removed depending on the wound condition. If the fascia had not fused with the surrounding tissue and instead built up a barrier, the CSF would penetrate the skin as it did pre-operationally. Thus, if there was no sign of skin breakage, the tension sutures could be safely removed one after the other at approximately 20 days post-operation. We hypothesize that there might be cell signals inside the CSF that encourage soft tissue growth^29^.

Regarding our surgery procedure, further details should be provided: (1) only a small amount of fascia latawith respect to the dural defect should be harvested in the repair surgery; however, the amount harvested for packing should be least 50% larger than the maximum area of CSF on sagittal MR imaging. For example, if the maximum area of the CSF is 40 cm^2^, the size of the fascia lata should be at least 15 cm × 4 cm to ensure that the fascia can reach the lateral walls after compression suturing; (2) before fascia lata packing, the lateral walls of the fragile pseudomembrane should be scraped until fresh bleeding would occurs to ensure sufficient blood supply; (3) the skin located around the breakout point of the pseudomeningocele should be removed to ensure wound healing; (4) tension-reducing sutures should be used to ensure the blood supply of the mid-line incision; (5) when performing compression suturing, the entrance and exit points of the needle should be located 6 cm away from the mid-line to ensure that the lateral walls of the pseudomeningocelearebrought sufficiently close to each other. At the same time, the suture should penetrate and secure the implanted fascia lata; otherwise, it could be displaced. (6) Close attention to the wound condition is necessary, and any ischaemic changes or signs of infection, such as swelling or secretion, should be addressed when they first appear.

During the follow-up period, no complications or recurrence was reported among our patients, except for some pain at the donor site and cervical pain induced by the pressure suture. Initially, 1 patient who underwent pre-operative radiation therapy, subsequently wound nonunion and infection were discovered. Since radiation exposure is known to affect wound healing ability, pre-operative radiation therapy was considered a contraindication, and we continue to search for better treatment protocols for these patients. For those with symptomatic pseudomeningocele and with short life expectancy, management may include consecutive antibiotics and conservative tumour-related treatment. The major limitation of this study was the small number of patients, which made it difficult to arrive at a strong, convincing result. Unfortunately, few patients are seen for spine surgery in our institution. Consequently, only 9 cases were collected over 10 years. A multi-centre study should be conducted to confirm that this method is convenient and effective.

Once the dura are invaded by metastatic or recurrent tumours, repairing ability is certainly compromised, and subsequent repair efforts become more difficult. Wound healing is also affected by the poor nutritional status which is induced by chemotherapy. Thus, even after complete repair of the dura, PMs cannot be completely avoided in certain cases. These cases are excellent candidates for fascia lata packing and tension suturing treatments I.

Our investigation shows that PMs can be successfully eliminated together with significant improvement of symptoms. The conclusion was made based on careful clinical observations and on statistically significant findings, although the sample size of this study is small. Nevertheless, our surgical protocols can be used by other clinicians. With more experience from use of the protocol, patients can be better served with better outcomes.

## Methods

### General information

All methods included in this report were carried out in accordance with our national guidelines and regulations. This study was approved by the Institutional Review Board of the First Affiliated Hospital of Shantou University Medical College, the First Affiliated Hospital, Sun Yat-Sen University and Guangzhou Medical University. All patients were above 18 years old, and the informed consent for study participation and for the publication of their images/data in an online open access publication was signed by themselves.

This study is a retrospective cohort study of 9 symptomatic pseudomeningocele patients treated with fascia lata packing and tension suturing between January 2008 and January 2018 in a single institution. The operations were performed by different teams under the same operation guidance. Data were collected and analysed by an attending surgeon and a consultant. All patients had failed conservative treatment before consulting for surgery. Patients were collected according to the following indications: (1) previous posterior cervical spinal tumour resection with CSF leakage and pseudomeningocele development; (2) CSF leakage that could not be stopped by extension of the drainage duration, suturing of the drainage exit in the skin or lumbar cistern drainage; (3) presentation with symptoms for 3 weeks, including postural headache and central neurological fever(if the wound had been penetrated, however, immediate surgery was offered); (4) pseudomeningocele confirmed on MRI; and () follow-up for more than 1 year. The exclusion criteria included (1) those who had contraindications for secondary surgery; (2) those who had severe complications after the first operation, such as neurological injury caused by intramedullary bleeding or dermatitis induced by post-operative radiation therapy, which would affect the wound union, or those who had a short life expectancy; (3) those who had abnormal liver and kidney function or long-term hypoproteinaemia, which would affect the wound union; and (4) those who had contraindications for fascia lata transplant surgery, such as an infection located at the donor site, pre-existing bacteraemia or pre-existing malignant tumour skin invasion.

### Operative technique

**Figure 3:**
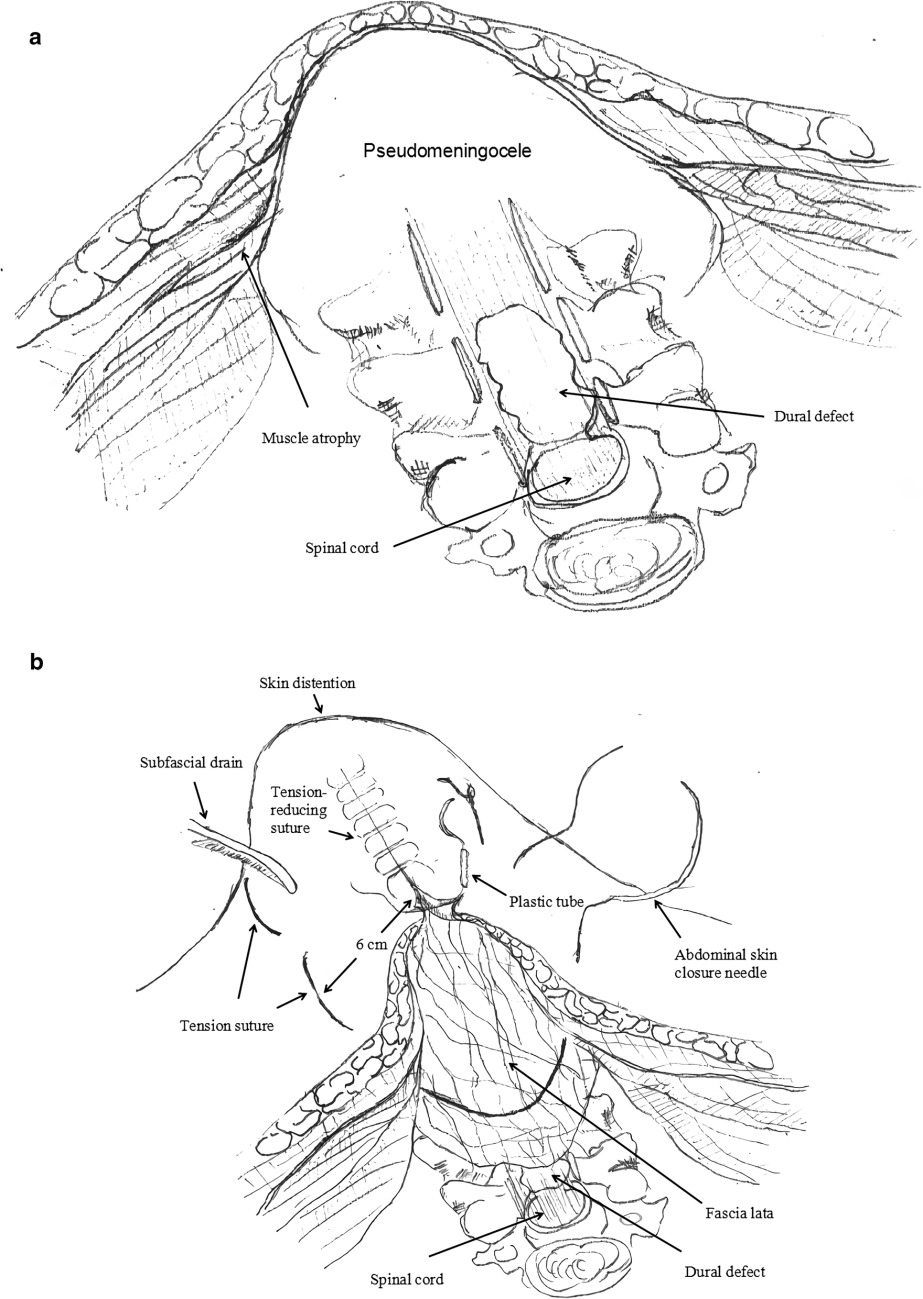

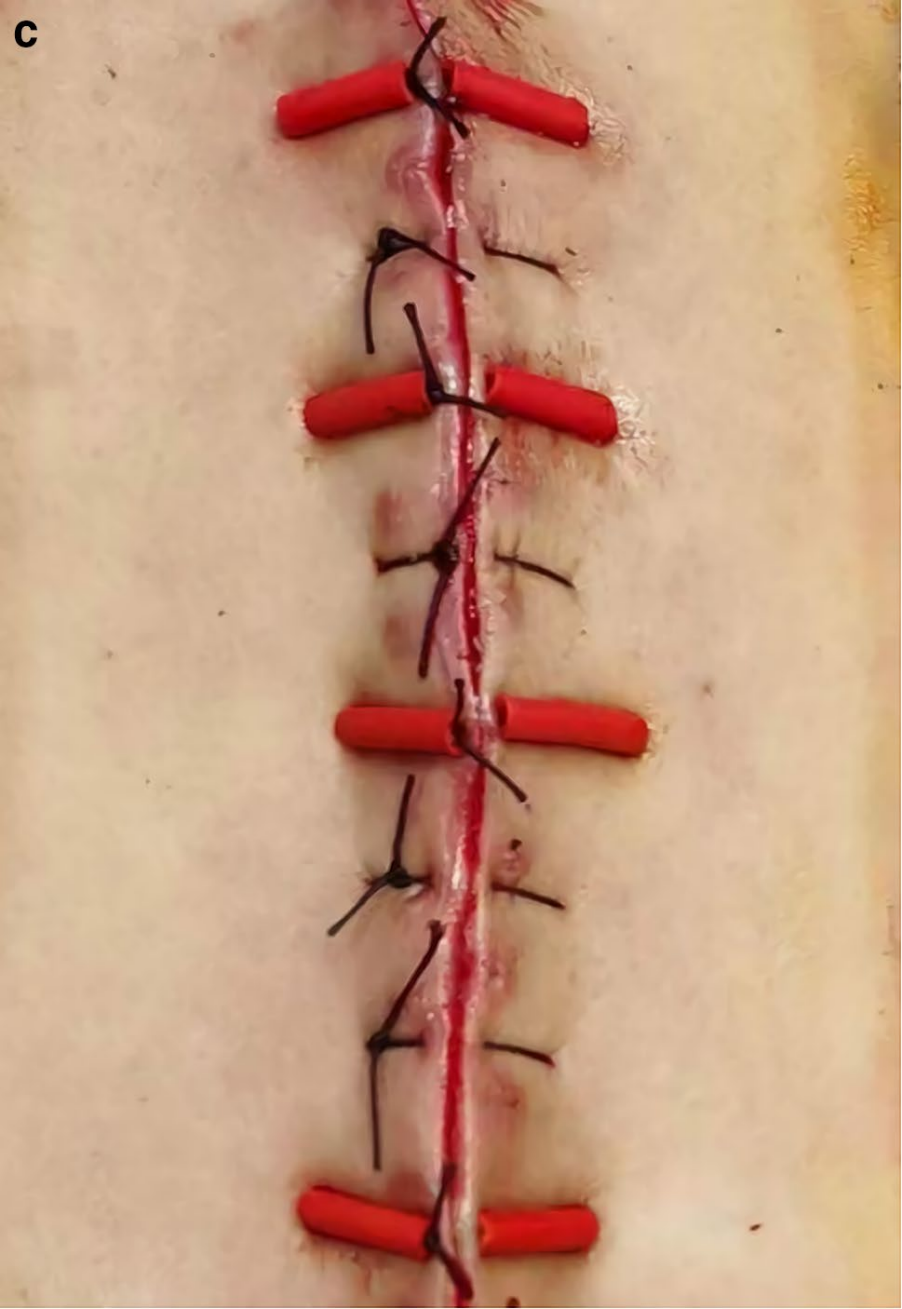
Operation diagram. (**a**) Formation of the pseudomeningocele. (**b**) Fascia lata was packed into the pseudomeningocele cavity and fixed by a compression suture. Then, the cervical posterior soft tissues were compressed and evaluated. (**c**) Post operative photograph of tension reducing suture.

Each patient was placed in the prone position, and a Mayfield device was fixed to the head. A mid-line incision was made to the cervical skin. After the CSF was absorbed, the smooth pseudomembrane surface of the fibrous capsule could be observed. The inflammatory tissue of the pseudomembrane was scraped until fresh bleeding was observed. The fascia lata was harvested from the lateral thigh through ha 10–15 cm incision. The area of fascia harvested to be packed into the pool was 50% larger than the maximum area of the pseudomeningocele on sagittal MR imaging. By using an abdominal skin closure needle, the fluid pool of the posterior neck was closed with tension sutures, with the insertion point and outlet point placed approximately 6 cm from the mid-line to ensure that the lateral walls of the pseudomeningocele would be brought sufficiently close to each other. At the same time, the suture was also threaded through the fascia to secure it. After one subfascial drainage tube was placed, the original wound was closed with a conventional tension-reducing simple interrupted suture. The technique is standard incisional skin sutures except 2 pieces of plastic tube were sutured on the wound surface, the plastic tubes could be considered as the elastic device offering buffer (Fig. 3). During the operation, a large quantity of normal saline was used to wash the operative field to prevent infection.

### Post-operative management

The drainage tube was left in place without suction. A bubble-like plastic container was connected to the drainage tube and maintained in an expanded state. This connected the drainage tube was subjected to positive pressure (whereas a bag would connect the tube to standard pressure). The more drainage fluid was found inside the container, the more positive the pressure would be. When the drainage was less than 50 ml per day, the container and tube were removed, and the drainage skin exit was sutured. Patients were recommended to lie on one side to avoid placing pressure on the wound. After 14 days, the mid-line suture was removed, and the patients were allowed to walk. The tension and drainage site sutures were removed approximately 20 days post-operation depending on the wound condition. The tension sutures were removed one after another. The first suture was removed 3 days after the removal of the mid-line suture, and then the wound condition was carefully monitored. If the wound expanded, the other suture was left in place; otherwise, the remaining suture was removed 3 days later. Daily dressing was necessary for these wounds.

Infection was prevented by antibiotics. Before surgery, a microbiologist was consulted, and antibiotics were given until wound union was observed. Typically, ceftriaxone or amoxicillin was chosen for the ability to pass through the blood–brain barrier. When there was suspicion of pre-operative intra-cranial infection, antibiotics were given at the discretion of the microbiologist. Clinical biological markers, such as CRP, ESR and blood count, were conducted every 3 days to monitor the inflammation.

### Clinical evaluation

Chief complaints, clinical history, physical examination, diagnosis and operative time were recorded. Clinical outcomes and wound union time were also recorded. On the MR images, the treatment was considered successful if: (1) the largest area of the pseudomeningocele was significantly reduced and (2) no transparent gap could be observed between the implanted fascia and surrounding tissue. The largest area of the pseudomeningocele on sagittal MRI sections was recorded pre- and 1 year post-operatively and analysed by the LEADTOOLS Medical Imaging system.

### Statistical analysis

Statistical analysis was performed with SPSS (version 18 for Windows, IBM). Data are presented as the means ± standard deviations (SDs). A Q–Q plot was used to determine if the data approximated a normal distribution. The paired t test was generally applied for normally distributed data of continuous variables (largest pre- and 1-year post-operative area of the pseudomeningocele on sagittal MRI sections), *P* < 0.05 (two-tailed) was considered significant, and the confidence interval was 95%.
